# Short-Term Effects of Conventional Chest Physiotherapy and Expiratory Flow Increase Technique on Respiratory Parameters, Heart Rate, and Pain in Mechanically Ventilated Premature Neonates: A Randomized Controlled Trial

**DOI:** 10.3390/healthcare12232408

**Published:** 2024-11-30

**Authors:** Jacqueline Carla de Macedo, Clarice Rosa Olivo, Viviani Barnabé, Eduardo Dati Dias, Íbis Ariana Peña de Moraes, Iolanda de Fátima Lopes Calvo Tibério, Celso Ricardo Fernandes de Carvalho, Beatriz Mangueira Saraiva-Romanholo

**Affiliations:** 1 Instituto de Assistência Médica Ao Servidor Público Estadual, Hospital do Servidor Público do Estado de São Paulo, São Paulo 04039-000, Brazil; jacqueline.macedo@gmail.com (J.C.d.M.); clarice.olivo@fm.usp.br (C.R.O.); vivianibarnabe@gmail.com (V.B.); beatriz.msaraiva@fm.usp.br (B.M.S.-R.); 2 Faculty of Medicine, University City of São Paulo, São Paulo 03071-000, Brazil; 3 Laboratory of Experimental Therapeutics, School of Medicine, University of São Paulo, São Paulo 01246-903, Brazil; iolanda.tiberio@fm.usp.br; 4 Department of Physiotherapy, Santa Catarina State University, Florianópolis 88080-350, SC, Brazil; 5 Postgraduate Program in Rehabilitation Sciences, Faculty of Medicine, University of São Paulo, São Paulo 05360-160, Brazil; edudati@gmail.com (E.D.D.); cscarval@usp.br (C.R.F.d.C.); 6 Department of Physiotherapy, Federal University of Juiz de Fora Campus Governador Valadares, Governador Valadares 35020-360, MG, Brazil

**Keywords:** newborn, premature infant, mechanical ventilations, physical therapy techniques

## Abstract

**Background/Objectives**: Prematurity is a leading cause of infant mortality and mechanical ventilation increases respiratory complication risk. The effects of secretion removal techniques in premature infants remain a topic of ongoing debate. The aim of the study is to compare two secretion removal techniques in premature infants on mechanical ventilation; **Methods**: The participants were randomized into conventional chest physiotherapy (CPT; *n* = 22) or expiratory flow increase technique (EFIT; *n* = 21) groups. Each participant completed four sessions on consecutive days with a minimum of one and a maximum of two sessions per day. We assessed peripheral oxygen saturation (SpO_2_), heart (HR) and respiratory rates (RR), tidal volume (VT), and pain levels at specific time points: before the intervention, immediately after the intervention, 5 min after the intervention, and 10 min after the intervention. The partial Eta squared (ŋ_p_^2^) was reported to measure the effect size; **Results**: HR and RR increased post-intervention in both groups (*p* < 0.001; HR ŋ_p_^2^ = 0.51; RR ŋ_p_^2^ = 0.38); post hoc comparisons showed EFIT group decreased RR from the first to last session (*p* = 0.045). Both groups exhibited increased VT and SpO_2_ in all sessions (*p* < 0.001; VT ŋ_p_^2^ = 0.40; SpO_2_ ŋ_p_^2^ = 0.50). The EFIT group had higher SpO_2_ values (*p* = 0.013; ŋ_p_^2^ = 0.15) and lower pain scores (*p* < 0.001; ŋ_p_^2^ = 0.46) post-intervention compared to CPT; **Conclusions**: CPT and EFIT resulted in similar effects on short-term respiratory parameters and heart rate; however, EFIT had advantages, including lower RR, higher SpO_2_, and reduced pain, suggesting it may be a more effective, comfortable neonatal respiratory treatment.

## 1. Introduction

Annually, around 15 million babies are born prematurely worldwide, and this rate is increasing in most countries. Prematurity is the leading cause of infant mortality [1]. However, the rise in prematurity has been accompanied by significant improvements in perinatal care, leading to a notable increase in infant survival rates, especially among those with very low birth weights admitted to neonatal intensive care units (NICUs) [2]. Advanced care during hospital stays involves multiple invasive procedures to enhance neonatal survival. Unfortunately, these procedures are often painful and can negatively impact quality of life as well as neuropsychomotor development [3,4].

In NICUs, premature infants are at high risk of developing respiratory complications and frequently require mechanical ventilation for support [5,6]. This procedure involves intubation and the insertion of an artificial airway, which can stimulate increased secretion production and worsen gas exchange, thereby impairing the mobilization and expectoration of secretions [5]. To address this, secretion removal techniques are employed by a multidisciplinary team to assist in clearing secretions, forming an integral part of the care for intubated patients [6,7]. These techniques have been used in clinical practice for decades and are traditionally considered a part of respiratory physiotherapy [8,9].

Airway clearance techniques are typically described as “conventional” or “contemporary”, with their effectiveness having been studied both individually (the effect of each technique in isolation) and in combination (the use of one or more techniques simultaneously). Conventional airway clearance techniques rely on gravitational forces or shock waves applied to the chest wall, such as postural drainage, chest compression, or vibration [10,11]. On the other hand, contemporary (or unconventional) techniques, like the expiratory flow increase technique (EFIT), are based on the principle of expiratory flow variation [12]. EFIT, originally developed for premature infants, consists of manual compression that focuses on synchronizing chest wall-abdominal movements, with the goal of inducing a slow expiratory flow at low lung volumes [12,13].

While respiratory physiotherapy has demonstrated benefits in invasively ventilated adults with pneumonia—such as increased tidal volume that may aid in secretion clearance and improve lung compliance [14]—evidence regarding its effectiveness in neonates remains conflicting [15]. Conventional chest physiotherapy (CPT) techniques aim to enhance secretion clearance while minimizing risks and avoiding undesirable effects in this vulnerable population [16,17]. Additionally, evidence indicates that CPT remains the primary focus of physiotherapy intervention in NICUs for mechanically ventilated patients, supporting its use as an effective strategy for secretion clearance in this setting [18].

A recent systematic review evaluating the effects of chest physiotherapy in preterm infants with respiratory distress syndrome found that all studies measuring peripheral oxygen saturation (SpO_2_) reported significant intragroup improvements, regardless of the technique used. However, while vibration and percussion techniques were applied in all CPT studies, the methods varied. Results regarding respiratory rate (RR) and hospitalization time were inconsistent, with some studies showing increases, others reductions, and some reporting no change. Despite these inconsistencies, a significant improvement in respiratory system compliance was observed following the intervention [6]. Undesirable effects of CPT include stress, pain, clinical instability, behavioral disorganization, and the inappropriate use of energy reserves in preterm infants [19,20].

Given that EFIT involves less patient manipulation, we hypothesize that it induces less stress and pain, potentially reducing the cardiorespiratory response and improving ventilation. A single prospective study [21] investigated EFIT, specifically examining the incidence of post-extubation atelectasis and brain injury in newborns. The findings indicate that post-extubation atelectasis is uncommon in newborns treated with EFIT, and chest physiotherapy does not appear to increase the risk of brain injury beyond the rates typically observed in newborns with respiratory failure. However, the author emphasizes the urgent need for randomized controlled trials to further evaluate the efficacy and safety of EFIT. Notably, no studies have yet assessed the effects of EFIT on cardiovascular and respiratory parameters in neonates. Therefore, the current study aims to compare the short-term effects of two secretion removal techniques—CPT and EFIT—on respiratory parameters, heart rate, and pain levels in mechanically ventilated premature neonates.

## 2. Materials and Methods

This was a prospective randomized and controlled trial with two arms. The current study complied with the Declaration of Helsinki, and the Research Ethics Committee of the General Hospital approved the protocol (protocol 13/2008). Patient caregivers or the legally responsible guardian signed an informed consent form, and the trial was registered at ClinicalTrials.gov (NCT03159039). No medical or multidisciplinary assistance was modified during the study.

### 2.1. Participants

Preterm infants admitted to the intensive care unit of a general hospital were evaluated for eligibility. The inclusion criteria were age (<35 gestational weeks or 259 days since the first day of the child’s mother’s last menstrual period); having >72 h of life and <7 days of life; being under mechanical ventilation; clinically and hemodynamically stable; having a birth weight ≤ 1500 g being characterized as very low birth weight; an inspired oxygen fraction (FiO_2_ ≤ 0.6); an inspiratory pressure (Pinsp) > 25 cmH_2_O, and a medical prescription for respiratory physiotherapy. The exclusion criteria included infants presenting severe congenital malformation, associated syndromes, persistent pulmonary hypertension, intracranial hemorrhage, or coagulopathies.

### 2.2. Sample Size (n)

Sample size was calculated based on the mean difference in means for equal-sized groups and was calculated using an Altman nomogram based on a study with similar characteristics and population where the variables were of quantitative interest. A sample size of 40 preterm infants was estimated as the number needed to provide a 90% power to detect an increase of 3.45 ± 3.37 breaths per minute 15 min post intervention, with an alpha level of 5% [7].

### 2.3. Allocation and Randomization

**Figure 1:**
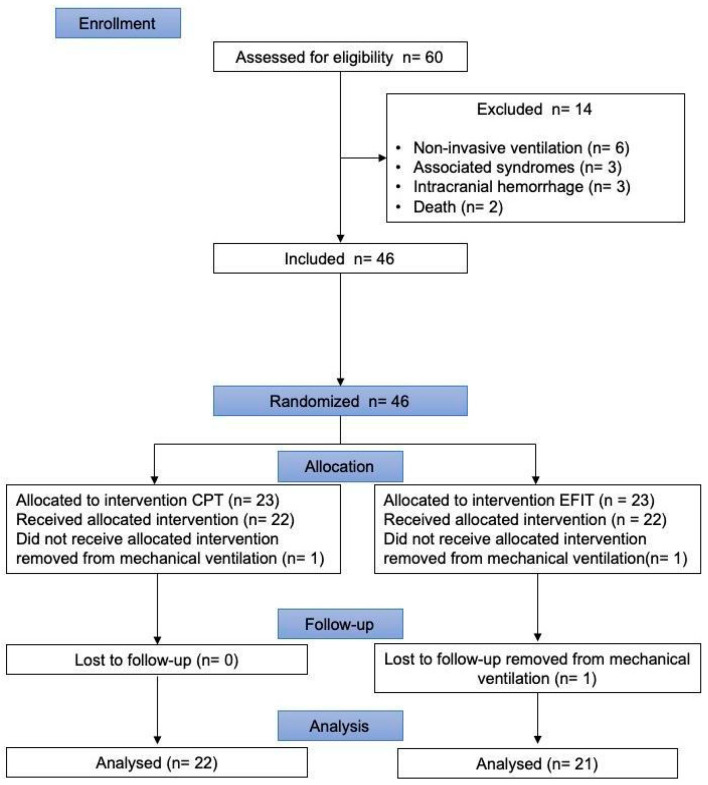
Flow of participants through the randomized controlled trial.

The eligible participants were randomly allocated to one of the following groups: the CPT or EFIT groups (Figure 1). Randomization was performed using a simple computer-generated randomization sequence obtained in (www.randomization.com, accessed on 7 September 2015) by one investigator who was not involved in the assessments or the patient’s treatment. The allocation was performed using numbered, sealed, opaque envelopes prepared for every participant. Each envelope corresponded to one of the groups and was chosen by the caregiver after the baseline measurements that included anthropometric, clinical, and ventilatory data [22].

### 2.4. Baseline Characteristics

Data were obtained from medical records and included gestational age at birth (in weeks), age at study (in days), weight (in grams), sex, delivery mode, medical diagnosis, medication use, and mechanical ventilation parameters. In addition, lung auscultation, respiratory rate, oxygen pulse oximetry, and the radiological chest pattern were also evaluated.

### 2.5. Study Design

After randomization, the infants were included in the CPT or EFIT group and the presence of pulmonary secretions was identified. The intervention was performed during consecutive days of hospitalization in the neonatal intensive care unit, totaling four sessions for each participant. Outcomes were assessed before and 5 and 10 min after the intervention. The interventions were carried out from September 2015 to May 2017.

### 2.6. Outcomes

Clinical data assessment: The infants received airway clearance techniques (either the CPT or the EFIT) for 10 min. Before, immediately after, and 5 and 10 min after the techniques, the following outcomes were evaluated: peripheral oxygen saturation (SpO_2_), cardiac and respiratory rates, tidal volume, and pain level. The heart rate (HR) and SpO_2_ were measured using a digital oximeter (DX 2010-Dixtal Biomedica, Manaus, Brazil). The RR, defined as the number of breaths per minute, was measured using a mechanical ventilator. The tidal volume (VT, in mL) was measured in milliliters (mL) using a mechanical internal ventilation sensor. The presence of pain was evaluated using the neonatal infant pain scale (NIPS), a validated pain scale for infants [23]. NIPS scores include questions related to crying, facial expression, the position of the legs and arms, and the sleep/wake state. The NIPS maximum score is 7 points, and pain is considered present when the sum of the points is ≥3 [23].

### 2.7. Interventions

**Figure 2:**
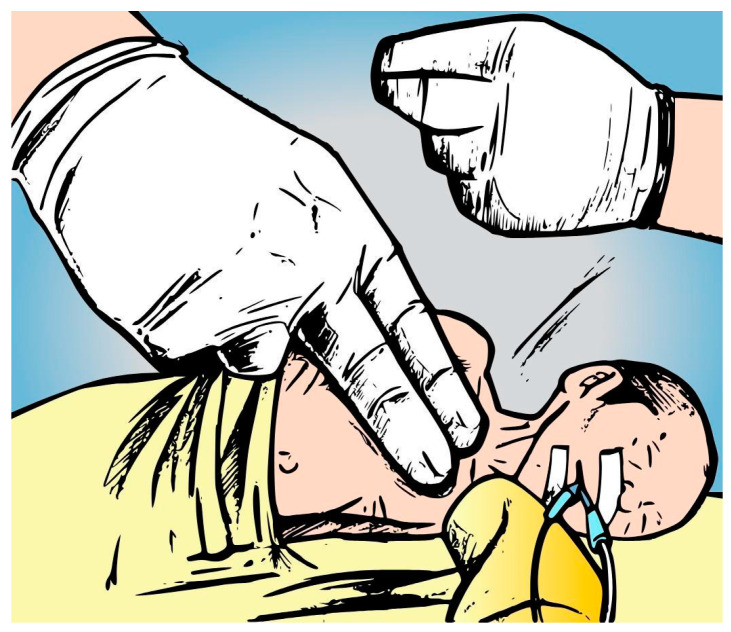
Conventional airway clearance (CPT). This figure was created by the authors specifically for this publication.

The CPT included a lateral postural drainage position (five minutes each side) associated with manual chest wall vibration and compression during the expiratory phase. During chest physiotherapy, all patients were monitored with continuous pulse oximetry. (Figure 2).

**Figure 3:**
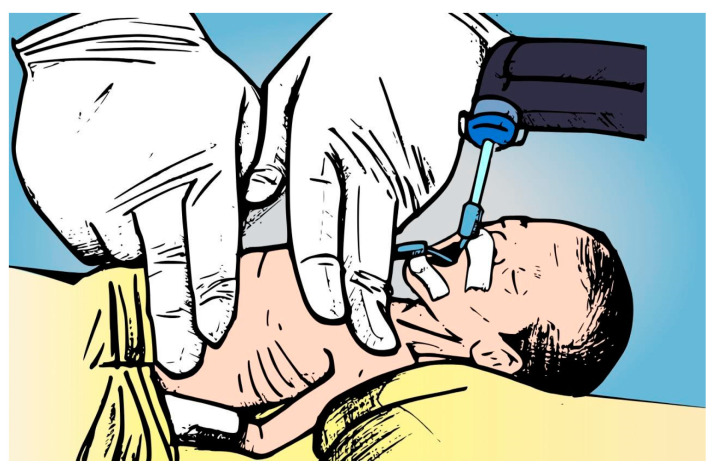
Expiratory flow increase technique (EFIT). This figure was created by the authors specifically for this publication.

The EFIT consists of synchronous thoracic-abdominal movement generated by the hands of the physiotherapist at the beginning of the expiration. One hand was placed on the chest wall (or index and middle fingers), and the other on the abdominal area. Mild pressure was applied on the chest wall toward the abdomen stabilized at the end of the expiratory phase until near the residual volume. The pressure was sustained for 2 s, and the procedure was repeated five to ten times per minute, over a period of 10 min (Figure 3). Finally, when there was a need, gentle suction was performed in both interventions to remove mucous secretions within the endotracheal tube for a maximum time of 15 s.

In these maneuvers, especially with premature infants, adaptations are necessary to accommodate their small and delicate anatomy. Rather than using the whole hand, therapists use just the index and middle fingers on the chest, usually near the sternum or lower rib cage, to perform both techniques. This finger-based method minimizes pressure and allows precise control over the force applied, reducing the risk of over-compression on fragile lung and chest structures [10].

### 2.8. Data Analysis

For the continuous independent variables, gestational age at birth, age at study, weight and parameters of pulmonary ventilatory support, a Student’s *t* test was used to compare groups. For the categorical independent variables, sex, delivery mode, medical diagnosis, medications, and ventilatory mode, a chi-square test was performed to compare groups.

The dependent variables, HR, RR, SpO_2_, VT, and NIPS, are presented as mean and standard deviation and were submitted to MANOVA for 2 Groups (CPT and EFIT) by Session (S1 to S4), by Moment (M1: Rest; M2: After intervention; M3: 5 min after intervention; M4: 10 min after intervention) with repeated measures in the last two factors. The Least Significant Difference (LSD) was used as the post hoc test.

The graph data are presented as mean and standard error. The partial Eta squared (ŋ_p_^2^) was reported to measure the effect size and interpreted as small (effect size > 0.01), medium (effect size > 0.06), or large (effect size > 0.14) [24]. The statistical package used was SPSS, version 26.0, and we considered *p*-values < 0.05 as significant.

## 3. Results

In total, 43 participants were assessed. The demographic data are shown in Table 1, demonstrating that the groups were homogeneous.

### Dependent Variables

**Figure 4:**
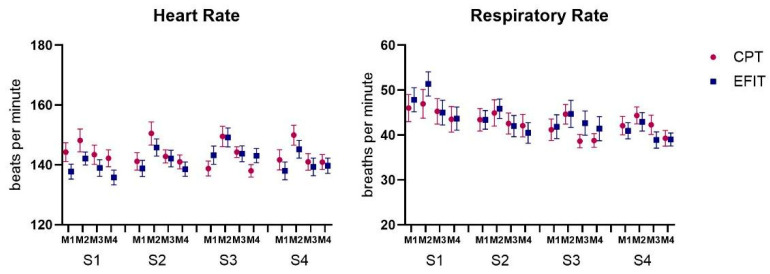
Representation of mean and standard error of heart rate and respiratory rate of both Groups and all Sessions (S1 to S4) and Moments (M1: Rest; M2: After intervention; M3: 5 min after intervention; M4: 10 min after intervention). CPT: conventional airway clearance; EFIT: expiratory flow increase technique.

MANOVA revealed a significant effect for Group (F_5, 34_ = 7.03; *p* < 0.001, ŋ_p_^2^ = 0.50; Wilks’ λ = 0.491) and Moment (F_14, 25_ = 50.29; *p* < 0.001, ŋ_p_^2^ = 0.96; Wilks’ λ = 0.034) and interactions were found for Session by Moment (F_14, 25_ = 7.18; *p* < 0.001, ŋ_p_^2^ = 0.80; Wilks’ λ = 0.199). Separate follow-up repeated measures (RM-ANOVAs) for HR, RR, SpO_2_, VT, and NIPS (Figure 4) are reported in the paragraphs below.

Considering HR (Figure 4), ANOVA revealed a mean effect of Moment (F_3, 114_ = 40.18; *p* < 0.001, ŋ_p_^2^ = 0.51), showing that HR was higher at M2 (M = 147 bpm) in both Groups and all sessions when compared to M1 (M = 140 bpm; *p* < 0.001), M3 (M = 142 bpm; *p* < 0.001), and M4 (M = 140 bpm; *p* < 0.001).

For RR (Figure 4), ANOVA revealed a mean effect of Moment (F_3, 114_ = 23.97; *p* < 0.001, ŋ_p_^2^ = 0.38), showing that RR was higher at M2 (M = 45.7 bpm) in both Groups and all sessions when compared to M1 (M = 43.4 bpm; *p* < 0.001), M3 (M = 42.3 bpm; *p* < 0.001), and M4 (M = 41.1 bpm; *p* < 0.001). Post hoc comparisons revealed that only the EFIT group presented a decrease in RR from S1 (M = 46.9 bpm) to S4 (M = 40.6 bpm; *p* = 0.045).

**Figure 5:**
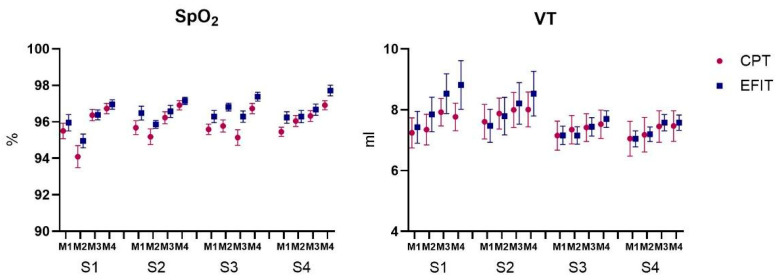
Representation of mean and standard error of peripheral oxygen saturation (SpO2) and tidal volume (VT) of both Groups and all Sessions (S1 to S4) and Moments (M1: Rest; M2: After intervention; M3: 5 min after intervention; M4: 10 min after intervention). CPT: conventional airway clearance; EFIT: expiratory flow increase technique.

Considering the VT, ANOVA revealed a mean effect of Moment (F_3, 114_ = 25.78; *p* < 0.001, ŋ_p_^2^ = 0.40), showing that VT was higher at M4 (M = 7.9 mL) in both Groups and all sessions when compared to M1 (M = 7.2 mL; *p* < 0.001), M2 (M = 7.4 mL; *p* < 0.001), and M3 (M = 7.8 mL; *p* < 0.001). Interactions were also found for Session by Moment (F_9, 342_ = 2.72; *p* = 0.035, ŋ_p_^2^ = 0.06), indicating an increase in VT from M1 (M = 7.2 mL) to M4 (M = 7.9 mL; *p* < 0.001) in all sessions and both groups (Figure 5).

For SpO_2_ (Figure 5), ANOVA revealed a mean effect of Group (F_1, 38_ = 6.8; *p* = 0.013, ŋ_p_^2^ = 0.15), showing that the EFIT group (M = 96.5%) had a higher SpO_2_ than the CPT group (M = 95.9) in all sessions and moments. A mean effect was also found for Moment (F_3, 114_ = 39.25; *p* < 0.001, ŋ_p_^2^ = 0.50), showing that SpO_2_ was higher at M4 (M = 97.0%) in both Groups and all sessions when compared to M1 (M = 95.8%; *p* < 0.001), M2 (M = 95.6%; *p* < 0.001), and M3 (M = 96.3%; *p* < 0.001). Session by Moment interactions were also found (F_9, 342_ = 8.49; *p* < 0.001, ŋ_p_^2^ = 0.18), showing an increase in SpO_2_ from M1 to M4 (*p* < 0.001) in all Sessions and both Groups.

**Figure 6:**
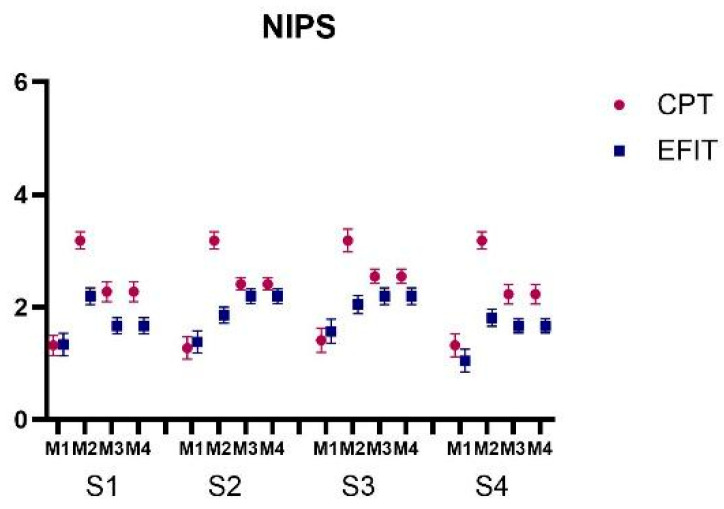
Representation of mean and standard error of neonatal infant pain scale (NIPS) of both Groups and all Sessions (S1 to S4) and Moments (M1: Rest; M2: After intervention; M3: 5 min after intervention; M4: 10 min after intervention). CPT: conventional airway clearance; EFIT: expiratory flow increase technique.

Lastly, for NIPS (Figure 6), ANOVA revealed a mean effect of Group (F_1, 41_ = 35.86; *p* < 0.001, ŋ_p_^2^ = 0.46), indicating that the EFIT group (M = 1.79) had a lower NIPS score than the CPT group (M = 2.31) in all sessions and moments. Mean effects of Moment (F_2, 40_ = 182.0; *p* < 0.001, ŋ_p_^2^ = 0.90) and Moment by Group interaction (F_2, 40_ = 53.8; *p* < 0.001, ŋ_p_^2^ = 0.72) and Session by Moment interaction (F_6, 36_ = 3.07; *p* = 0.016, ŋ_p_^2^ = 0.33) were also observed, showing that the CPT group had a higher NIPS score in all Sessions than the EFIT group at M2 (M = 3.18; M = 1.97; *p* < 0.001, respectively), M3 (M = 2.36; M = 1.92; *p* < 0.001), and M4 (M = 2.36; M = 1.92; *p* < 0.001).

## 4. Discussion

The results of this study demonstrate that the application of CPT and EFIT in premature infants on mechanical ventilation induces similar acute effects on respiratory parameters and heart rate. However, EFIT resulted in less pain and more favorable responses in peripheral oxygen saturation and respiratory rate compared to CPT. These findings suggest that EFIT elicits a lower pain response, making it a potentially less stressful intervention for this population.

Few studies have evaluated the benefits of secretion removal techniques in neonates, and the results are often conflicting [6,15,17]. Additionally, most studies focus on patients with bronchiolitis, with limited research on those under mechanical ventilation. This study is pioneering in comparing secretion removal techniques applied to premature neonates on invasive mechanical ventilation. This is crucial, as these techniques are routinely used in NICUs despite the limited supporting evidence.

In the current study, both CPT and EFIT produced similar effects on the respiratory parameters of premature infants, leading to improvements in SpO_2_ post-intervention. However, EFIT consistently achieved higher SpO_2_ values throughout all assessment moments. Almeida et al. [25] observed similar improvements in ventilated infants with acute pulmonary obstruction, attributing the changes to bronchial secretion clearance and enhanced gas exchange. These findings align with studies demonstrating the efficacy of EFIT in infants with recurrent wheezing [26] and neonates with acute viral bronchiolitis (AVB) [27]. Furthermore, a systematic review of chest physiotherapy for AVB in pediatric patients indicated that slower techniques effectively improve symptoms and reduce the AVB clinical severity score [28]. Similarly, Mishra et al. [29] showed that the prolonged slow expiratory technique improved SpO_2_ and resolved pulmonary congestion in neonates with congenital pneumonia, emphasizing EFIT’s potential to optimize gas exchange and respiratory outcomes.

These results, though derived from diverse populations, support the findings of our study, which is the first to highlight the benefits of EFIT in premature infants on mechanical ventilation. This is particularly relevant for such patients, who exhibit high clinical demands and fragile conditions. Techniques that minimize physiological disturbance provide greater benefits in this vulnerable group [7,16,30].

Both CPT and EFIT were associated with transient cardiopulmonary response in our study. For example, there was a trend toward an average increase in HR post-intervention, although values remained within normal limits and returned to baseline within 10 min. Notably, EFIT caused a smaller increase in HR compared to CPT. Some studies have reported favorable cardiovascular outcomes with CPT in preterm newborns under invasive mechanical ventilation [7,30] and noninvasive ventilation [31,32], suggesting it is safe when monitored appropriately. One study reported that HR increased after CPT but remained stable following the expiratory flow increase technique EFIT in post-extubation premature infants, suggesting that EFIT may be a less stressful intervention [32]. In our study, the lack of significant differences in HR between the techniques could reflect the expertise of the professionals, who skillfully minimized physiological disturbances during the procedures. Similarly, Tavares et al. [33] investigated the acute effects of conventional respiratory physiotherapy in 30 preterm newborns with respiratory distress syndrome. Their findings revealed a transient increase in HR and pain levels immediately following the intervention, with all parameters returning to baseline within 15 min, indicating no prolonged adverse effects.

A trend of increased RR was observed after both techniques, but RR returned to baseline in subsequent moments. Over the course of EFIT sessions, RR decreased, accompanied by an inversely proportional improvement in SpO_2_. In contrast, CPT-induced RR increases may be attributed to repositioning, which can awaken neonates and increase arousal [34,35]. These findings are consistent with studies suggesting that techniques requiring less positional adjustment, like EFIT, are better tolerated.

A randomized controlled trial by Gomes et al. [36] assessed the effectiveness of three respiratory physiotherapy techniques in 30 infants with AVB, demonstrating that CPT and EFIT significantly reduced clinical scores compared to upper airway suction alone. Additionally, EFIT caused less pain than CPT in our study. This aligns with a systematic review by Zanelat et al. [37], which noted that techniques like endotracheal suctioning and vibratory compression often cause pain in neonates, emphasizing the importance of selecting gentler interventions [38].

The safety and efficacy of EFIT have been highlighted in other studies, such as those by Demont et al. [21] and Antunes et al. [32], which reported fewer systemic effects and lower complication rates compared to CPT. Our findings support the use of less stressful techniques like EFIT to improve outcomes for premature neonates, given their vulnerability to clinical complications such as hypoxemia, bradycardia, and respiratory fatigue.

As anticipated, both CPT and EFIT demonstrated significant immediate effects on VT in this study. This finding is consistent with Flenady et al. [10], who, in their systematic review, noted that respiratory physiotherapy could transiently improve lung mechanics, including VT. However, they also pointed out the inconsistent results across trials and emphasized the limited evidence regarding the safety and applicability of these techniques in current clinical practices.

EFIT demonstrates significant advantages over CPT, including less pain, better tolerance, and improved oxygenation, making it a promising alternative for premature neonates on mechanical ventilation. These results underscore the importance of refining clinical protocols to incorporate less invasive, more effective techniques to support the recovery of this vulnerable population.

Our findings hold significant clinical implications, given the vulnerability of neonates to various complications. Elevated metabolic rates in neonates can lead to hypoxemia and progress to bradycardia, driven by myocardial hypoxia and acidosis. This risk is exacerbated by their immature lungs, which predispose them to early-onset respiratory fatigue and, ultimately, respiratory failure [12]. To address these challenges, several protocols have been developed to guide healthcare professionals in delivering neonatal care that is less painful and more humane [39,40,41]. For example, the Newborn Individualized Developmental Care and Assessment Program aims to mitigate the adverse effects experienced by premature neonates in hospital settings [42]. However, these protocols do not currently include secretion removal techniques.

We believe our findings could directly inform the refinement of neonatal care protocols by incorporating secretion removal techniques that minimize stress, pain, and metabolic expenditure, thereby promoting early recovery. In line with this, the American Academy of Pediatrics (2016) emphasizes the fundamental right of every child, regardless of age, to have their pain reduced and prevented [43].

Some important considerations should be noted regarding our study, we did not quantify mucus volumes suctioned, as the focus was on comparing secretion drainage techniques and its effects on respiratory parameters, as well as heart rate and pain. Although precise mucus measurement is rare in clinical practice, observational monitoring tailored to patient needs is common. The literature emphasizes individualized suctioning based on respiratory status, with studies like Clifton-Koeppel [44] and Cordero et al. [45] advocating against routine suctioning in favor of clinical evaluation. While some research, such as Ackerman and Gugerty [46], reports differences in sputum weights between techniques, the clinical relevance remains unclear, especially for premature infants with very low birth weight. Further research is needed to standardize mucus quantification and assess its significance in neonatal care.

Also, auscultation as a clinical variable in neonates is limited by subjectivity, clinician experience, and the challenges of interpreting faint lung sounds in small, rapidly breathing infants. These factors make auscultation less reliable for standardized assessments, highlighting the need for objective tools in neonatal respiratory evaluations [47,48].

This study has some limitations. First, the study did not account for evolving guidelines, such as the current recommendations on protective peak inspiratory pressure (PIP) for premature infants, which emphasize lower PIP values (15–20 cm H_2_O for very low birth weight [VLBW] infants) to minimize lung injury; incorporating these parameters in future research could enhance its applicability [49]. Second, publication was delayed due to various factors, and while this did not affect data validity, more timely dissemination could have improved its relevance to contemporary neonatal care. Third, the study population predominantly consisted of VLBW infants (90%), limiting generalizability, as the intervention could potentially benefit premature newborns up to 2500 g [50]. Fourth, mucus suction and auscultation were not quantified, which could have provided valuable data on the efficacy of secretion removal techniques; future studies should include methods for quantifying mucus volume and standardizing auscultation. Fifth, arterial blood gas data were unavailable due to routine service procedures that did not align sampling times with intervention periods, limiting physiological insights. Sixth, closed suction systems are not widely used in Brazil’s NICUs due to higher initial costs, limited access, required staff training, and the need for advanced ventilators, which are not universally available, further influencing the study’s applicability. Seventh, key outcomes such as mortality, duration of mechanical ventilation, length of ICU and hospital stays, and weaning were not collected, limiting the evaluation of the broader impact of these interventions. Eighth, limited comparable studies in the literature hinder contextualization of the findings, highlighting the need for further research. Lastly, the short follow-up period, necessitated by logistical challenges and caregiver resistance, precluded the assessment of long-term outcomes; however, the study successfully identified lower impacts on stress indicators. To address these limitations, future studies should include more diverse populations, extended follow-up periods, mucus quantification, traditional outcome measures, arterial blood gas data, and evaluations of closed suction system applications. Additionally, comparing various airflow variation techniques could help refine neonatal respiratory care protocols and improve our understanding of secretion removal strategies.

## 5. Conclusions

The results of our study demonstrate that, in the short-term parameters, the CPT and the EFIT induced similar effects in respiratory parameters and heart rate. However, EFIT showed additional benefits, such as decreased RR at the last session, lower pain, and higher SpO_2_ than CPT, suggesting it may be a more effective and less uncomfortable option for neonatal respiratory treatment.

## Figures and Tables

**Table 1:** Demographic data.

Variables	CPT	EFIT	*p*-Value
	*n* = 22	*n* = 21	
	Mean ± SD	Mean ± SD	
Gestational age birth (weeks)	30.38 ± 1.99	30.82 ± 1.84	0.464
Age at study (in days)	5.65 ± 1.17	5.64 ± 1.20	0.965
Weight (grams)	1275.00 ± 171.47	1292.57 ± 156.37	0.728
Sex	*n* (%)	*n* (%)	
Male	11 (50)	14 (66.66)	0.213
Female	11 (50)	7 (33.33)	
Delivery mode	*n* (%)	*n* (%)	
Cesarean section	14 (63.63)	13 (61.90)	0.578
Vaginal birth	8 (36.36)	8 (38.09)	
Medical diagnosis—RDS	*n* (%)	*n* (%)	
Grade I	6 (27.27)	5 (23.80)	0.641
Grade II	14 (63.63)	12 (57.14)	
Grade III	2 (9.09)	4 (19.04)	
Medications	*n* (%)	*n* (%)	
Received sedative—Fentanyl	21 (95.45)	18 (85.71)	0.286
Received antibiotic	16 (72.72)	16 (76.19)	0.536
Surfactant doses	*n* (%)	*n* (%)	
Zero	6 (27.27)	4 (19.04)	0.714
One	12 (54.54)	14 (66.66)	
Two	4 (18.18)	3 (14.28)	
Ventilatory mode	*n* (%)	*n* (%)	
Assisted pressure-controlled ventilation	13 (59.09)	14 (66.66)	0.422
Support pressure	9 (40.90)	7 (33.33)	
Parameters of pulmonary ventilatory support	Mean ± SD	Mean ± SD	
Inspiratory pressure	17.0 ± 1.5	16.99 ± 1.4	0.865
Positive end-expiratory pressure	5.45 ± 0.5	5.31 ± 0.4	0.283
Inspired fraction of oxygen	35.68 ± 9.5	32.29 ± 5.2	0.156

CPT: chest physiotherapy technique; EFIT: expiratory flow increase technique; RDS: respiratory distress syndrome. Data presented as Mean ± Standard Deviation (SD) or number (percentage); independent *t* test for gestational age at birth, age at study and weight (between groups); chi-square for sex, Delivery mode, medical diagnosis, medications and ventilatory mode (between groups).

## Data Availability

The original data presented in the study are openly available in Mendeley Data [51].
